# Dkk1 Regulates Ventral Midbrain Dopaminergic Differentiation and Morphogenesis

**DOI:** 10.1371/journal.pone.0015786

**Published:** 2011-02-11

**Authors:** Diogo Ribeiro, Kristina Ellwanger, Désirée Glagow, Spyridon Theofilopoulos, Nina S. Corsini, Ana Martin-Villalba, Christof Niehrs, Ernest Arenas

**Affiliations:** 1 Section of Molecular Neurobiology, Department of Medical Biochemistry and Biophysics, Karolinska Institute, Stockholm, Sweden; 2 Division of Molecular Embryology, DKFZ-ZMBH Alliance, German Cancer Research Center, Heidelberg, Germany; 3 Division of Molecular Neurobiology, German Cancer Research Center, Heidelberg, Germany; Center for Regenerative Therapies Dresden, Germany

## Abstract

Dickkopf1 (Dkk1) is a Wnt/β-catenin inhibitor that participates in many processes during embryonic development. One of its roles during embryogenesis is to induce head formation, since *Dkk1*-null mice lack head structures anterior to midbrain. The Wnt/β-catenin pathway is also known to regulate different aspects of ventral midbrain (VM) dopaminergic (DA) neuron development and, *in vitro*, Dkk1-mediated inhibition of the Wnt/β-catenin pathway improves the DA differentiation in mouse embryonic stem cells (mESC). However, the *in vivo* function of Dkk1 on the development of midbrain DA neurons remains to be elucidated. Here we examined *Dkk1^+/−^* embryos and found that Dkk1 is required for the differentiation of DA precursors/neuroblasts into DA neurons at E13.5. This deficit persisted until E17.5, when a defect in the number and distribution of VM DA neurons was detected. Furthermore, analysis of the few *Dkk1^−/−^* embryos that survived until E17.5 revealed a more severe loss of midbrain DA neurons and morphogenesis defects. Our results thus show that Dkk1 is required for midbrain DA differentiation and morphogenesis.

## Introduction

Dickkopf 1 (Dkk1) is a secreted glycoprotein belonging to the Dkk family, which consists of four members (Dkk-1, -2, -3, -4). All Dkks share two conserved cystein-rich domains separated by a linker region [1], [2]. Dkk1 is a known Wnt/β-catenin pathway inhibitor: it binds to Lrp5/6, preventing its interaction with the Wnt protein and disrupting the Wnt-induced Frizzled-Lrp6 complex formation necessary for signal transduction [3], [4], [5]. Dkk1 can also block the Wnt/β-catenin pathway by inducing Lrp6 endocytosis in the presence of Kremen proteins [6]. During embryonic development *dkk1* is first expressed in *Xenopus* in the Spemann organizer of the early gastrula, and in mouse in the anterior visceral endoderm, anterior mesendoderm and foregut endoderm [1]. Dkk1 has been shown to have a major role in inducing head formation: injection of *dkk1* mRNA in *Xenopus* embryos leads to anteriorized embryos with big heads and enlarged cement glands; together with a dominant-negative mutant of the BMP2/4 receptor, *dkk1* mRNA is also able to induce secondary axes with complete heads [1]. Loss-of-function studies further confirm that Dkk1 is essential for head induction: *Xenopus* embryos injected with an anti-Dkk1 antibody [1] and *Dkk1* knockout mice [7] lack anterior head structures. The head-inducing activity of Dkk1 is mediated through the inhibition of the Wnt/β-catenin posteriorizing activity in early gastrula embryos [8], and in combination with the inhibition of BMP signalling [9]. Besides its role in anterior neural patterning, Dkk1 is involved in limb formation [10], [11], vertebral development [12], bone formation [13], [14] and bilateral eye induction [8]. Dkk1 has also been described to regulate cell proliferation and programmed cell death [7],[15],[16], and to have a role in diseases such as cancer [17] and Alzheimer's disease [18]. Consistent with its role *in vivo*, Dkk1 is able to induce neural differentiation from embryonic stem cells [19], [20], [21]. The majority of the Dkk1 effects result from a direct inhibition of the Wnt/β-catenin pathway, although Dkk1 can modulate gastrulation movements independently of β-catenin through activation of the Wnt/Planar Cell Polarity (PCP) pathway [22], suggesting that Dkk1 is able to modulate two different branches of Wnt signaling.

The Wnt/β-catenin pathway is involved in several aspects of neural development [23], [24], and has been described to play an important role in ventral midbrain (VM) dopaminergic (DA) neuron development: Wnt1, the prototypical ligand of the Wnt/β-catenin pathway, has been shown to regulate midbrain development [25], [26], neurogenesis [27], proliferation of DA progenitors [28], [29] and differentiation [30]; the Lrp6 receptor has been described to be important for the onset of midbrain DA differentiation and morphogenesis [31], and β-catenin is necessary for the integrity of the VM neurogenic niche and the progression from progenitors to DA neurons [32]. However, we also found increased DA neuronal differentiation in *Wnt1* ^−/−^ and *Lrp6* ^−/−^ mouse embryonic stem cells (mESC) [33]. These effects were mimicked in wild-type mESC by treatment with Dkk1, indicating that Dkk1 promotes DA differentiation in vitro. In order to address whether Dkk1 plays a role in the development of ventral midbrain DA neurons *in vivo* we examined *Dkk1* deficient mice. Our results indicate that Dkk1 regulates the distribution and the number of DA neurons in the developing VM. Interestingly we found that Dkk1 is required for the differentiation of Nurr1^+^/TH^−^ DA precursors (radially migrating neuroblasts) into DA neurons. Our results thus identify Dkk1 as a new regulator of midbrain DA neuron development.

## Materials and Methods

### Animals

Animals were housed in specific pathogen free and light, temperature (21°C) and humidity (50–60% relative humidity) controlled conditions. Food and water were available ad libitum. Mice were mated overnight and noon of day of plug was taken as E0.5. *Dkk1^+/−^* mice [7] were maintained in C57BL/6 congenic genetic background. *Dkk1^+/−^* embryos were obtained heterozygote crosses and by heterozygote x wild type crosses. *Dkk1^+/−^* and *Dkk1^−/−^* embryos were compared to wild-type littermates. The procedures for performing all animal experiments were in accordance with the principles and guidelines of the ATBW (officials for animal welfare), Karolinska Institutet as well as with the German and Swedish law. The permits were reviewed by the Internal Animal Protection Commission of the German Cancer Research Center (DKFZ) and the experiments were approved by the administrative headquarter “Regierungspräsidium Karlsruhe” of the State Baden-Württemberg. The approval is based on a positive vote of an appointed state governmental ethical commission according to §15 of the German Animal Protection law (approved license numbers: G-108/05, A-08/05, DKFZ180 and DKFZ206). CD1 mice (Charles River) were housed, bred, treated and analyzed in accordance with the permit approved by the Swedish ethical committee “Stockholms Norra Djurförsöketiska Nämnd” (ethical approval numbers N154/06 and N145/09).

### *In situ* hybridization and Immunohistochemistry

For *in situ* hybridization (ISH), embryos were fixed overnight before being cryopreserved in 30% sucrose, frozen in OCT and coronally sectioned (14 µm) onto Superfrost slides. ISH for *Dkk1* was performed in fixed tissue as described [28], [34] with digoxigenin-labelled single-stranded RNA probes at 70°C, followed by incubation with alkaline phosphatase (AP)- coupled antibody and nitroblue tetrazolium (NBT) plus 5-bromo-4-chloro-3-indolyl phosphate (BCIP) (purple) substrates.

For immunohistochemistry (IHC), coronal sections (12–14 µm thick) were obtained after adjusting the angle. Sections were pre-incubated for 1 hour in blocking solution (PBS, 0.25% Triton-X 100 and 5% normal goat serum) followed by incubation at 4°C overnight with one or more of the following primary antibodies diluted in blocking solution: rabbit anti-TH (1∶300, PelFreeze), mouse anti-TH (1∶500, ImmunoStar), mouse anti- βIII tubulin (Tuj1;1∶1000, Promega), rabbit anti-Lmx1a (1∶500, gift from M. German), rabbit anti-Nurr1 (1∶250, Santa Cruz Biotech.), rabbit anti Ki67 (1∶500, Neomarkers), mouse anti-Islet1 (1∶100, Developmental Studies Hybridoma Bank), rabbit anti-Pitx3 (1∶500, gift from M. Smidt), rabbit anti-Wnt1 (1∶500, Abcam), rabbit anti-cleaved Caspase 3 (1∶100, Cell Signaling), mouse anti-Brn3a (1∶500, Millipore). After washing, slides were incubated for 1–2 hours at room temperature with the appropriate secondary antibodies: biotinylated (1∶400, Jackson Laboratories) or fluorophore conjugated (1∶700, AlexaFluor 555 and 488). TO-PRO1-iodide nuclear stain (1mM, 1∶200, Invitrogen) was performed for visualization of cells. Biotinylated secondary antibodies were visualized with the Vector Laboratories ABC immunoperoxidase kit, using 3-3′ diaminobenzidine tetrahydrochloride (DAB 0.5 mg/ml); endogenous peroxidase activity was quenched for 20 minutes with 5% H_2_O_2_ prior to pre-incubation with secondary antibody. When necessary, antigen retrieval was performed prior to incubation with primary antibody with a target retrieval solution (Dako). ISH and IHC photos were acquired with a Zeiss Axioplan2 microscope and collected with a Hamamatsu camera C4742-95 with the Openlab and Photoshop software. Confocal pictures were taken with a Zeiss LSM 5 EXCITER microscope.

### VM primary cultures

Ventral midbrains of E10.5 CD1 mice were dissected out in ice-cold PBS supplemented with 0.2% glucose, mechanically dissociated in serum-free N2 medium through flame-narrowed Pasteur pipettes and plated at a final density of 150,000 cells/cm^2^ in poly-D-lysine-coated plates. Cultures were treated with recombinant mouse Wnt3a (100ng/ml, R&D) and the equivalent volume of 0.1% bovine serum albumin (BSA) as a control. Treatment of cultures was initiated at the time of plating and cultures were incubated for 6 hours in N2 medium at 37°C in 5%CO_2_. Cells were lysed and total RNA was extracted using the RNeasy Mini Kit (Qiagen), 1 µg was treated with RQ1 RNase-free DNase (Promega, Madison, WI) and reverse transcribed using SuperScript II Reverse Transcriptase (Invitrogen) and random primers (Invitrogen) (RT^+^ reaction). Parallel reactions without reverse transcriptase enzyme were done as a control (RT^−^ reaction) and Sybr green real-time quantitative PCR assays were performed as previously described [35]. Expression levels were obtained by normalization with the value of the housekeeping gene encoding 18S rRNA (Ambion, Austin, USA) obtained for every sample in parallel assays. The primers sequence for *Dkk1* were as follows: Forward-TCAATTCCAACGCGATCAAGA; Reverse- GGCTGGTAGTTGTCAAGAGTCTGG.

### Cell Counts and Statistical Analyses

Cell counts were performed in every fifth 14 µm coronal midbrain section through the entire DA domain (SN and VTA, from rostral to caudal). Graphics show the average number of cells (somas or nuclei) stained with antibodies against TH, Lmx1a, Nurr1, Pitx3, Islet1, Brn3a, Caspase3 or Ki67 in the serial sections and the entire DA domain, for every embryo analyzed. Depending on the embryonic stage and staining, 3–8 *Dkk1^+/−^*embryos were counted per condition (see figure legends). Nuclear markers were counted using ImageJ. All the measurements were performed in coronal sections through the midbrain and the distances were measured in pixels using ImageJ. The ventral tegmental (VTA) height was determined by drawing a vertical line between the most dorsal and most ventral cells in the VTA. Results in text and the graphs are presented as mean ± standard error of the mean (s.e.m) for each genotype. Cell numbers were compared with a Student's t-test using GraphPad. (*) p<0.05, (**) p<0.01, (***) p<0.001.

## Results

### Dkk1 is expressed in the developing ventral midbrain

**Figure 1:**
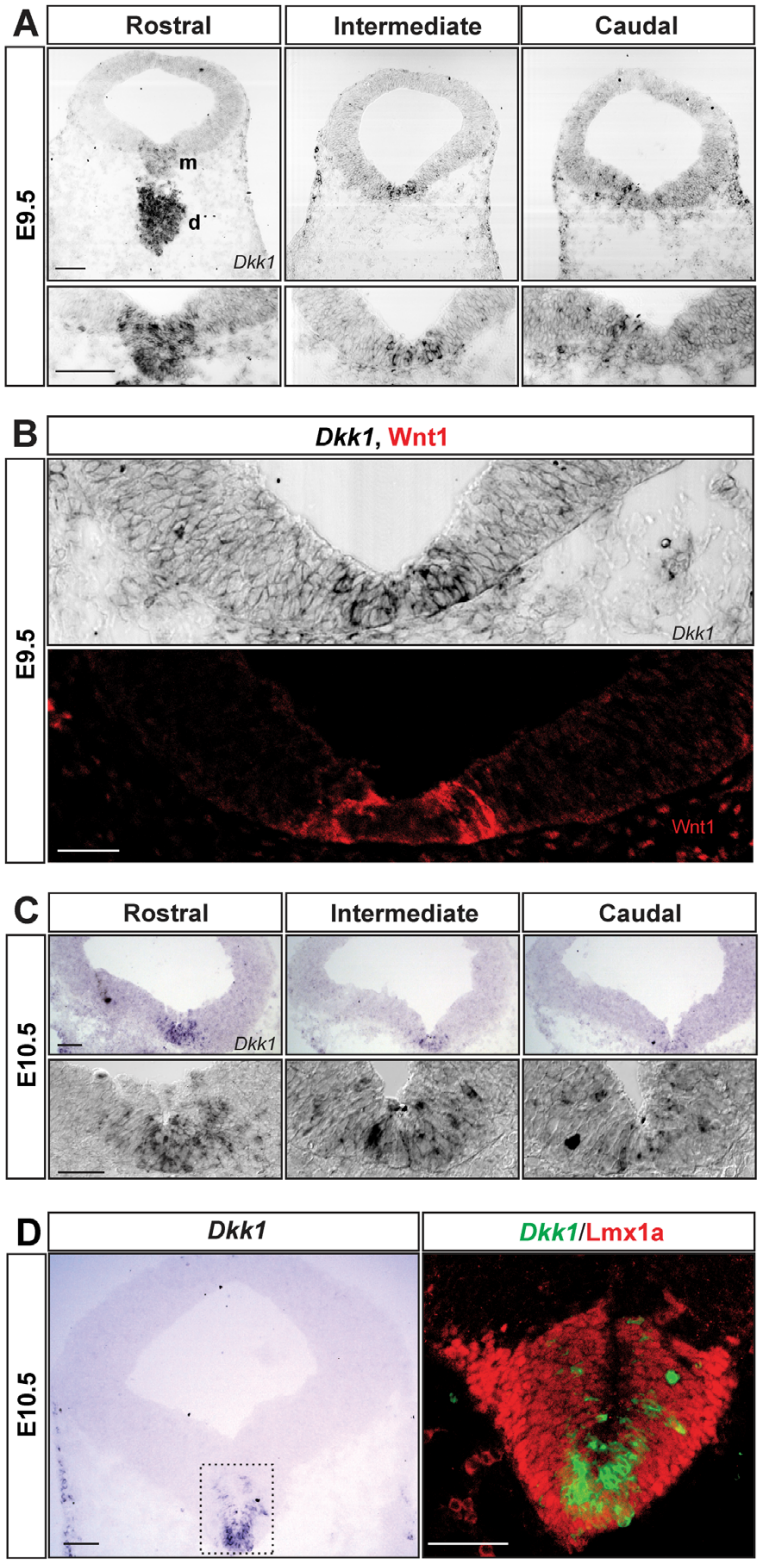
*Dkk1* is expressed in the ventral midbrain during the dopaminergic neurogenic period. (A) *In situ* hybridization for *Dkk1* on coronal sections at E9.5 revealed that *Dkk1 mRNA* is highly expressed in the diencephalon and in a salt-and-pepper pattern in the ventral midbrain, with higher expression rostrally and lower caudally. Scale bars = 100 µm. Abbreviations: **m-** midbrain, **d**- diencephalon. (B) Immunohistochemistry for Wnt1 on an adjacent section to the *Dkk1*-probed section shown in (A) revealed that Wnt1 protein and *Dkk1* mRNA are expressed in the VM at the same time and in a complementary anterior-posterior (not shown) and ventro-lateral manner (B). Scale bar = 50 µm. (C) At E10.5, *Dkk1* is expressed in the medial part of the floor plate in the ventricular, intermediate and marginal zones. Scale bars = 100 µm upper panels, 50 µm bottom panels (high magnification). (D) Imunohistochemistry for Lmx1a revealed that, at E10.5, *Dkk1* ^+^ cells are found in the medial part of the Lmx1a expression domain. Scale bars = 100 µm.

*Dkk1* is dynamically expressed in several areas of the developing central nervous system, including the mesencephalon [36], [37], but its expression in relation to DA neurons has not been examined. In order to characterize the spatial and temporal pattern of expression of *Dkk1* during the DA neurogenic period, we performed *in situ* hybridization in E9.5–13.5 mouse embryos. *Dkk1* was detected at significant levels in the VM at E9.5 and E10.5, but it was undetectable at E11.5–13.5. At E9.5, *Dkk1* was highly expressed in the ventral diencephalon and was expressed in a salt and pepper pattern in the VM, where it followed a rostro-caudal gradient with higher expression levels rostrally than caudally (Figure 1A). Interestingly, cells expressing *Dkk1* were found both in the lateral part of the floorplate, which expresses Wnt1, and in the medial part of the floorplate (Figure 1B). At E10.5, *Dkk1* mRNA was detected in the three layers of the developing VM (ventricular, intermediate and marginal zones, Figure 1C). At this stage, the midbrain expression of *Dkk1* was restricted to the medial part of the floorplate, where it was highly expressed in cells adjacent to the midline (Figure 1C). In order to confirm the midbrain DA identity of the *Dkk1*-expressing cells at E10.5, we analyzed the expression of *Dkk1* relative to that of Lmx1a, a transcription factor expressed in the entire DA lineage [38]. Immunohistochemistry for Lmx1a on *Dkk1*-probed sections revealed that only a medial subpopulation of Lmx1a^+^ cells expressed *Dkk1* (Figure 1C). Shortly after, at E10.75, *Dkk1* was only weakly expressed rostrally and no expression was detected in intermediate and caudal levels (data not shown). *Dkk1* expression was not detectable after E10.75. The position of *Dkk1^+^* cells and the timing of *Dkk1* expression suggested that Dkk1 may work as a regulator of DA neurogenesis and/or DA differentiation. We therefore examine these two processes in *Dkk1* mutant mice.

### Midbrain DA neuron development is not affected in Dkk1^+/−^ mice at E11.5

**Figure 2:**
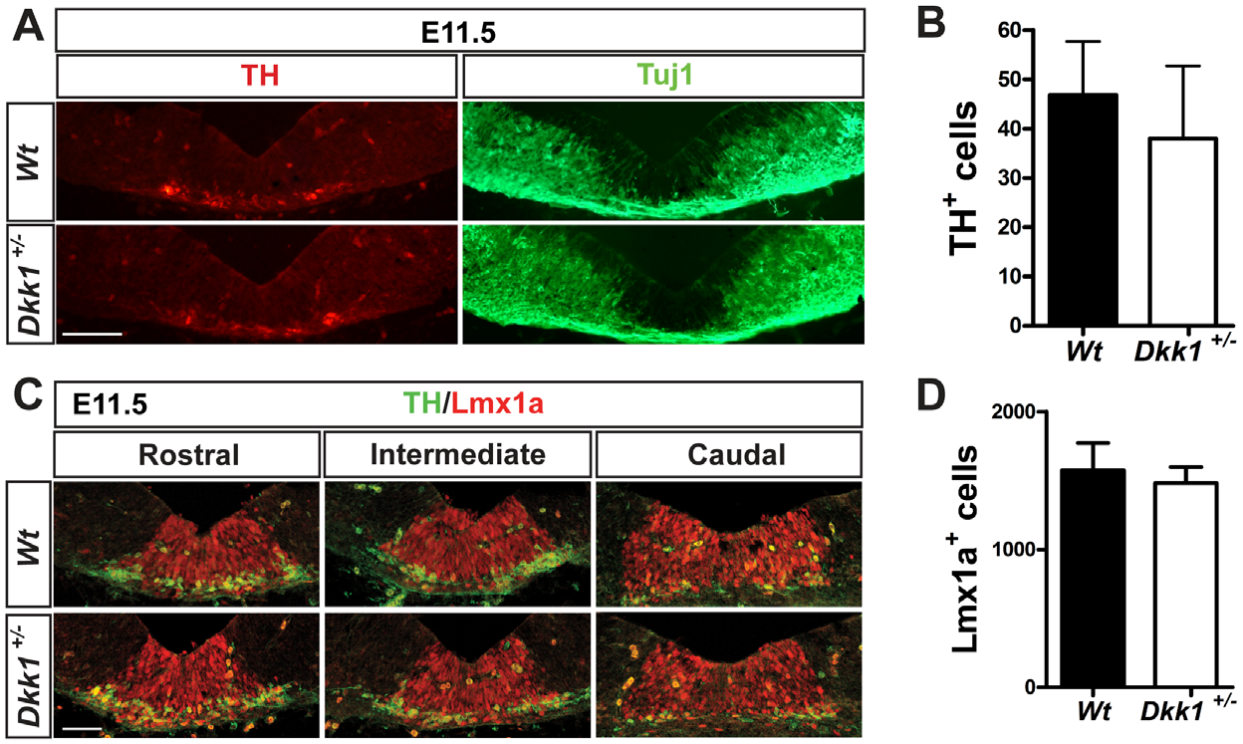
Initial dopaminergic neurogenesis is not affected in *Dkk1^**+/−**^* animals. (A) Immunohistochemistry for TH and TuJ1 in representative intermediate midbrain sections at E11.5. Scale bar = 50 µm (B) Quantification of the number of TH^+^ neurons in the VM revealed no differences in *Wt* and *Dkk1^+/−^* embryos (mean ± s.e.m- ***Wt*** **:** 46.8±10.9, N = 6; ***Dkk1^**+/−**^*** **:** 38±14.7, N = 5. (C) Immunohistochemistry for TH and Lmx1a in serial sections through the VM revealed no alteration in DA cells at E11.5. Scale bar = 50 µm. (D) No differences were observed in the number of Lmx1a^+^ cells at this stage (mean ± s.e.m- ***Wt*** **:** 1577±195.7, N = 3; ***Dkk1^**+/−**^*** **:** 1483±114.9, N = 3).

To address the function of *Dkk1* in midbrain development we examined *Dkk1^+/−^* and *Dkk1^−/−^* embryos. However, since *Dkk1^−/−^* mice showed a deletion of brain structures including the midbrain, we focused our analysis on *Dkk1^+/−^* embryos. At E11.5, when DA neurogenesis starts, we did not observe any significant difference in the numbers of TH^+^ cells (TH- tyrosine hydroxylase, the rate limiting enzyme in dopamine synthesis and a DA neuron marker) in heterozygous embryos compared with wild-type littermate controls (Figure 2A and 2B). Similarly, when we examined the expression of the early neuronal marker Tuj1, no differences were found (Figure 2A). To further examine whether other aspects of midbrain DA development were affected in the *Dkk1^+/−^* embryos we performed immunohistochemistry for Lmx1a and again found no differences between *Dkk1^+/−^* and *Wt* embryos in the number or distribution of progenitor cells at E11.5 (Figure 2C and D). These results indicated that the initial aspects of DA neuron development were not affected by the loss of one allele of *Dkk1*.

### Dopaminergic differentiation is impaired in Dkk1^+/−^ mutants

**Figure 3:**
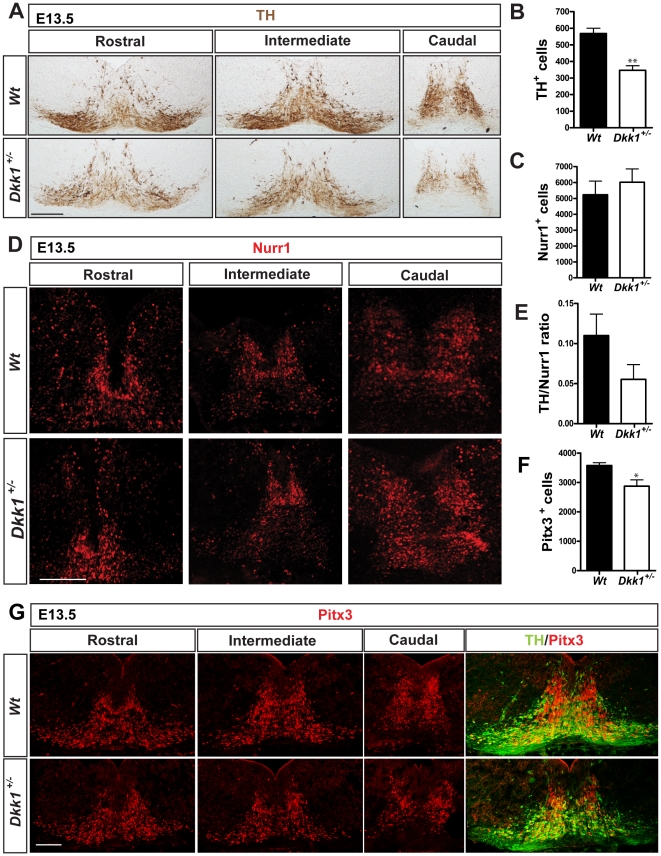
Dopaminergic neuron differentiation is disrupted in *Dkk1* mutants. (A) Immunohistochemistry for TH on midbrain serial coronal sections at E13.5. Scale bar = 100 µm. Quantification in (B) revealed a 40% decrease in the numbers of dopaminergic neurons in *Dkk1^+/−^* embryos when compared to *Wt* littermate controls (mean ± s.e.m- ***Wt*** **:** 568.3±31.7, N = 3; ***Dkk1^**+/−**^*** **:** 346.5±27.2, N = 8 p = 0.0015 ** unpaired t-test). (C, D) The Nurr1^+^ dopaminergic precursors are not affected in *Dkk1^+/−^* embryos (mean ± s.e.m- ***Wt*** **:** 5233±857.0 N = 2; ***Dkk1^**+/−**^*** **:** 6019±840.8 N = 3). Scale bar = 100 µm. (E) The TH/Nurr1 ratio indicated a differentiation deficit in the *Dkk1^+/−^* embryos (mean ± s.e.m- ***Wt*** **:** 0.109±0.027, N = 2; ***Dkk1^**+/−**^*** **:** 0.055±0.018, N = 3). (F, G) Expression of Pitx3 was reduced in *Dkk1^+/−^* embryos compared with wild-type littermates (mean ± s.e.m- ***Wt*** **:** 3574±99.02, N = 3; ***Dkk1^**+/−**^*** **:** 2874±216.4, N = 3, unpaired t-test p = 0.042*). Scale bar = 100 µm.

To determine whether DA neuron development was affected at later stages we performed immunohistochemistry for TH in *Dkk1^+/−^* E13.5 embryos, the time at which DA neurogenesis peaks in the VM. Interestingly we found a significant reduction (40%) in the number of DA neurons in the *Dkk1^+/−^* embryos compared to *Wt* (Figure 3A and 3B). In order to exclude the possibility of a general developmental delay we measured the crown-rump length in *Wt* and *Dkk1^+/−^* embryos and found no differences (Figure S1B). To determine if the observed phenotype was due to a general neurogenic defect we analyzed the expression of Tuj1 and saw no decrease between *Dkk1^+/−^* and *Wt* embryos (Figure S1A). Moreover, analysis of the expression of Islet1, which labels oculomotor neurons, and Brn3a, which labels the red nucleus neurons in the VM, revealed no difference between *Wt* and *Dkk1^+/−^* embryos at E13.5 (Figure S2), suggesting that the observed phenotype was specific. To determine whether the decrease in the number of TH^+^ cells was due to increased cell death we performed immunohistochemistry for active Caspase 3, which labels cells in apoptosis. However, very few Caspase3^+^ cells were detected in *Wt* and *Dkk1^+/−^* embryos, and no significant difference in the number of apoptotic cells was detected (Figure S3A). We next sought to determine which stage of DA differentiation was being affected by the absence of one *Dkk1* allele. The expression of Ki67 (a cell cycle marker) in the ventricular zone of the VM was not altered at E13.5, indicating that the DA progenitor pool was not affected (Figure S3B). Analysis of the expression of Nurr1, a marker of dopaminergic precursors (radially migrating neuroblasts) and neurons did not show any difference between *Dkk1^+/−^* and *Wt* embryos at E13.5 (Figure 3C and D). These results indicated that a normal number of postmitotic DA precursors are generated. We then examined the TH/Nurr1 ratio to measure the proportion of Nurr1^+^ cells that become TH^+^ cells. Interestingly, we found a decrease in the differentiation of DA precursors into TH^+^ neurons in *Dkk1* ^+/−^ embryos compared with *Wt* littermates (Figure 3E). Moreover, when the number of cells expressing the DA-specific transcription factor Pitx3 was examined, we found a small albeit significant reduction in the number of Pitx3^+^ cells at E13.5 (Figure 3F and G), confirming that *Dkk1* indeed regulates the DA differentiation of precursors into DA neurons.

### The DA differentiation defect persists at later stages in Dkk1^+/−^ mutants

**Figure 4:**
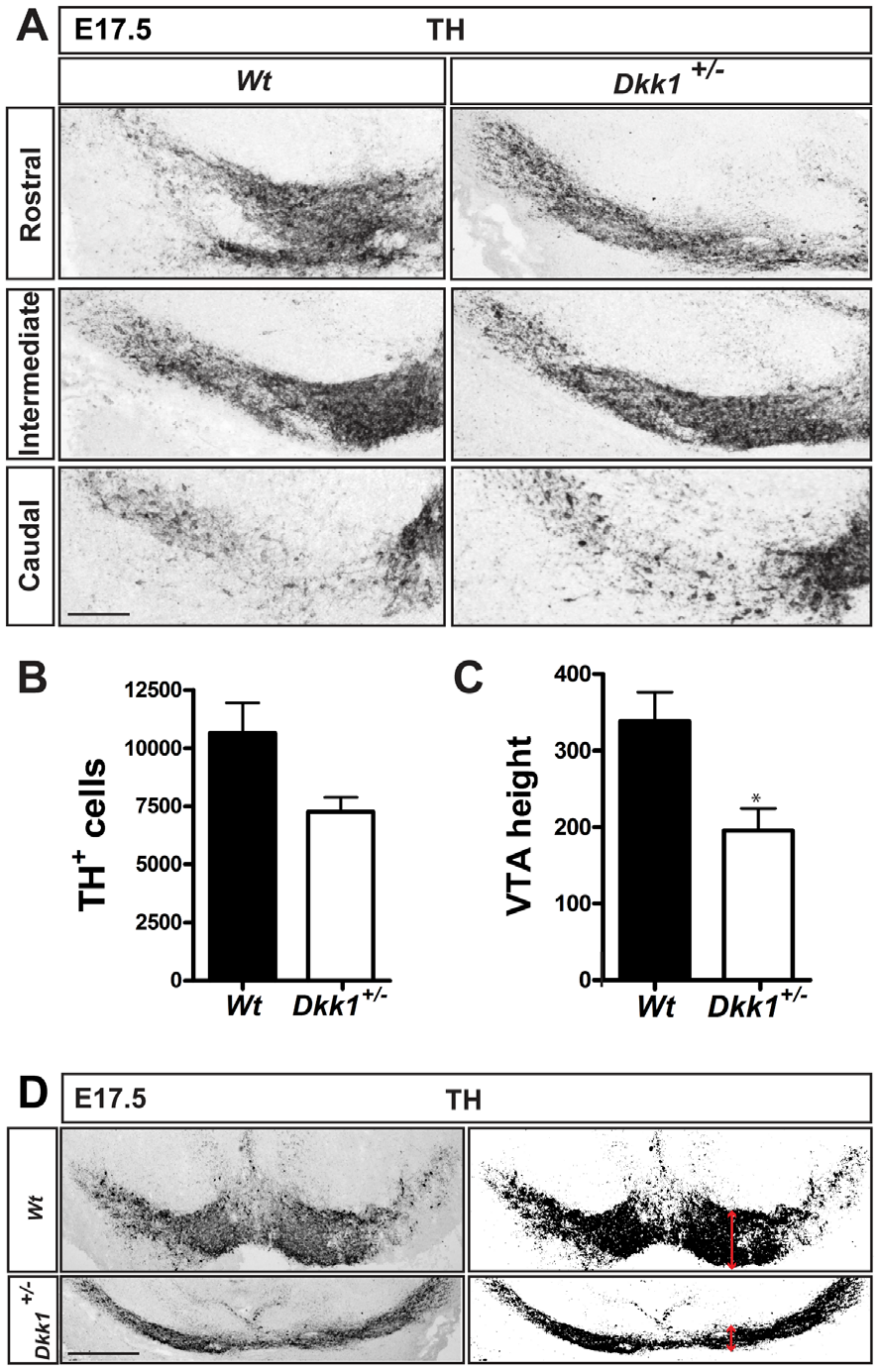
Dopaminergic differentiation deficit persists at later stages in *Dkk1* mutants. (A) Immunohistochemistry for TH at E17.5 in the VM revealed a decrease in the number of TH+ neurons and an alteration in their distribution. Scale bar = 100 µm. (B) A 30% decrease in the number of dopaminergic neurons was detected at this stage (mean ± s.e.m- ***Wt*** **:** 10640±1301 N = 4; ***Dkk1^**+/−**^*** **:** 7262±616.9 N = 3). (C) The abnormal distribution of TH^+^ cells was evident when the height of the VTA was measured (mean pixels ± s.e.m- ***Wt*** **:** 338.2±38.17 N = 5; ***Dkk1^**+/−**^*** **:** 195.3±28.90 N = 3, p = 0.041*, unpaired t-test). (D) The height of the VTA was measured in *Dkk1^+/−^* and *Wt* embryos using ImageJ. The heigth of the VTA domain is indicated by the vertical arrows in the right panels. Scale bar = 100 µm.

We next examined if the impairment in DA differentiation observed at E13.5 persisted at later embryonic stages and we analyzed the expression of TH in E17.5 embryos. Interestingly, we found a 30% decrease in the number of TH^+^ neurons in *Dkk1* ^+/−^ mice in comparison with *Wt* littermate controls (Figure 4A and 4B), indicating that the differentiation deficit does not recover during late embryonic stages. At E17.5, we also observed an abnormal distribution of TH^+^ DA neurons at different anterior-posterior levels in the VM (Figure 4A). To evaluate the extent of this phenotype we measured the height of the DA domain at the VTA level and found a significant decrease in *Dkk1* ^+/−^ embryos (Figure 4C and D). Interestingly, this phenotype was only evident in *Dkk1* ^+/−^ mice at E17.5. However, we also found that 20% of the E13.5 *Dkk1* ^+/−^ embryos showed exencephaly (not shown). Thus, our results indicate that in addition to a defect in DA differentiation, an alteration in VM cell distribution and morphogenesis also takes place in *Dkk1^+/−^* mice.

### VM morphogenesis and DA neuron development is severely disturbed in Dkk1^−/−^ mice

**Figure 5:**
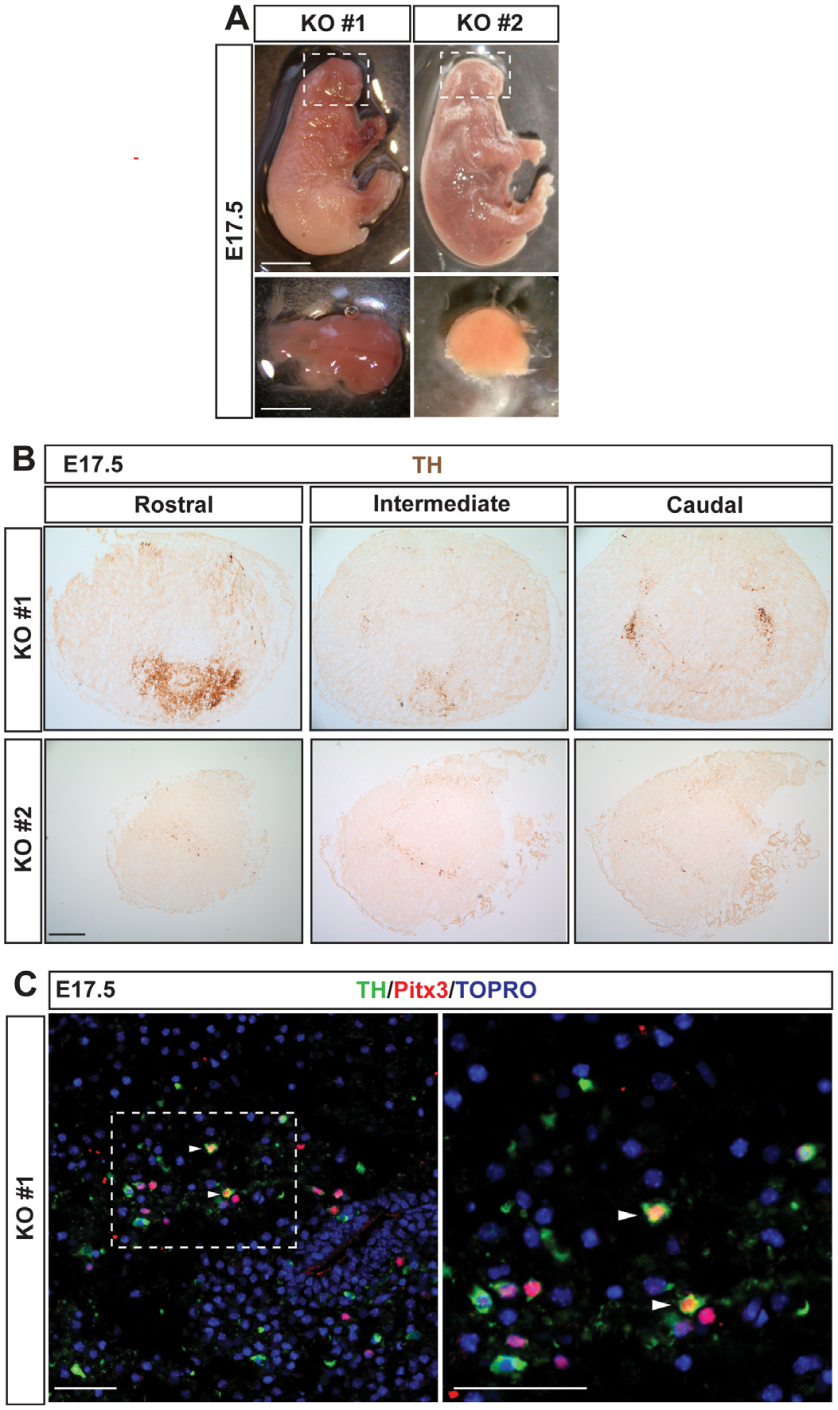
The morphogenesis and development of VM dopaminergic neurons is disturbed in *Dkk1^**−/−**^* embryos. (A) *Dkk1^−/−^* embryos at E17.5 revealed an absence of most facial and head structures. In some animals an incipient midbrain was still present (KO #1, dashed box) but in others there was no identifiable or absent midbrain (KO #2, dashed box). Scale bar = 0.5 cm. The remaining brain structures were dissected out (bottom panels, scale bar = 0.2 cm). (B) Immunohistochemistry for TH in midbrain sections of *Dkk1^−/−^* embryos revealed a decrease of aberrantly distributed dopaminergic neurons (upper panels) or an almost absence of dopaminergic neurons (bottom panels). Scale bar = 100 µm. (C) Double labeling of TH and Pitx3 revealed that only few of the TH^+^ cells were Pitx3^+^ (arrowheads). Scale bars: 50 µm.

In order to further characterize the function of Dkk1 in midbrain DA neuron development we analyzed the few E17.5 *Dkk1^−/−^* mice that we obtained. As expected, these embryos showed a strong phenotype which included a deletion of the most anterior head structures [7]. The level of midbrain deletion varied in the animals examined from embryos in which it was possible to identify a rudimentary midbrain, (Figure 5A, KO#1) to embryos with no identifiable or absent midbrain (Figure 5A, KO#2). In these embryos, immunohistochemistry for TH revealed either a severe decrease in the number of TH^+^ cells or an almost complete absence of DA neurons (Figure 5B). In the few *Dkk1^−/−^* mice in which TH^+^ cells were present, they were not found in their typical position and were very abnormally distributed forming clusters (Figure 5B). These findings indicated that Dkk1, directly or indirectly, is required for proper VM morphogenesis and cell distribution. Moreover, the TH^+^ cells found in some of the *Dkk1^−/−^* mice displayed an atypical morphology with very few or no projections (Figure 5C). Interestingly, only a few of these TH^+^ cells were found to express the midbrain specific transcription factor, Pitx3 (Figure 5C, arrowheads). These results thus indicate that while some midbrain tissue can still be formed in the absence of *Dkk1*, *Dkk1* is required for DA differentiation and midbrain morphogenesis.

## Discussion

The development of midbrain DA neurons proceeds in a tightly regulated fashion by several signaling pathways and transcription factors. Understanding the mechanisms responsible for the birth, differentiation and maintenance of these cells is of great interest to improve the generation and yield of DA neurons for cell-replacement therapies in Parkinson's disease. We previously showed that Wnt signaling is important for different aspects of midbrain DA neuron development [28], [30], [31], [39], and that Dkk1-mediated inhibition of the Wnt/β-catenin pathway improves the DA differentiation in mESC [33]. This led us to investigate whether Dkk1 plays a role in the development of midbrain DA neurons *in vivo*. Analysis of *Dkk1^+/−^* embryos showed that *Dkk1* is required for the differentiation of DA precursors into DA neurons. This defect was to some extent specific as Islet1^+^ motor neurons and Brn3a^+^ red nuclei neurons were not affected. Furthermore, analysis of the few *Dkk1^−/−^* embryos that survived until E17.5 revealed a decrease or a near absence of midbrain DA neurons and a severe defect in midbrain morphogenesis.

### *Dkk1* is expressed in a very precise temporal and spatial pattern during VM development

The expression of *Dkk1* was detected in the VM at E9.5 and its expression started to decrease at E10.5, just before the onset of DA neurogenesis. Interestingly, β-catenin expression and transcriptional activity, as assessed in the TOPGAL reporter mice, has been found to follow a similar spatial-temporal expression pattern [28]. Importantly, it has been described that *Dkk1* is a direct transcriptional target of β-catenin [40], [41], [42], [43] as part of a negative feedback loop. In agreement with this possibility, we hereby report that treatment of primary E10.5 VM cultures with Wnt3a, a known activator of Wnt/β-catenin signaling in this culture [28], acutely upregulates the expression of *Dkk1* (Figure S4). Combined, these results suggest that the activation of the Wnt/β-catenin pathway leads to the induction of *Dkk1* just before the birth of DA neurons, as an integral part of VM development. In support of this possibility, Wnt1 shows a similar temporal expression pattern compared to that of *Dkk1*, as it is highly expressed at E9.5. However, the spatial expression pattern of Wnt1 and *Dkk1* are slightly different, as they seem to form a complementary anterior-posterior and ventro-lateral gradients. While Wnt1 is expressed at higher levels in the posterior midbrain, in the lateral part of the floor plate, *Dkk1* is expressed at higher levels in the anterior midbrain, closer to the ventral midline. The spatial-temporal coordination of Wnt1 and *Dkk1* expression suggests that Dkk1, by inhibiting Wnt signaling until E10.5, regulates the transition from Wnt inhibition to activation in a region specific pattern, allowing proper differentiation of the midbrain DA neurons.

### Dkk1 controls the differentiation of migratory DA precursors/neuroblasts *in vivo*

Our study shows that Dkk1 is necessary for the proper development of midbrain DA neurons, as shown by the selective decrease in the number of DA cells in *Dkk1^+/−^* mice and the severe loss of DA neurons in *Dkk1^−/−^* embryos. Interestingly, we found that neither the number of proliferating progenitors in the ventricular zone nor the number of postmitotic DA precursors in the intermediate zone or the number of active Caspase 3^+^ cells in the VM was affected, indicating that DA progenitors are properly specified and that proliferation, survival or initial neurogenesis is not affected. However, when the differentiation of DA precursors was examined, we found a decrease in the numbers of TH^+^ and Pitx3^+^ cells at E13.5 without a concomitant decrease in the numbers of precursors (Nurr1^+^/TH^−^). This defect was maintained until E17.5, indicating that the loss of Dkk1 cannot be compensated at later stages of development. The precise mechanism by which deletion of *Dkk1* impaired DA differentiation remains to be elucidated. However, since we previously found that Dkk1 can block the activation of β-catenin and the Dvl phosphorylation shift induced by Wnt3a in DA cells [33], one possible explanation could be that excessive and/or premature activation of the Wnt/β-catenin signaling may prevent the proper differentiation of DA precursors in *Dkk1^+/−^* embryos. In agreement with this possibility, we recently found that activation of Wnt/β-catenin signaling *in vivo* (by conditional deletion of β-catenin exon3 with Shh-Cre) results in the accumulation of DA progenitors, impaired DA differentiation and reduced number of midbrain DA neurons [44]. Thus, our results suggest that factors regulating the appropriate levels of Wnt/β-catenin signaling, such as Dkk1, are essential for the development of midbrain DA neurons. Interestingly, a balanced Wnt/β-catenin signaling seems to be of great importance not only for the development of the VM, but also for the proper development of other tissues. For instance, Dkk1 and Wnt3 are expressed in adjacent domains during gastrulation and interact genetically to induce head formation [45]. In this case, anterior head truncation of *Dkk1*-null mice can be ameliorated by removing one allele of *Lrp6* [12] or of *Wnt3a* [45]. Thus combined, all data currently available in the literature indicate that a balance between positive and negative regulators of Wnt/β-catenin signaling is required for proper neural development.

### Dkk1, morphogenesis and the PCP pathway

One additional finding in the *Dkk1^+/−^* embryos was the alteration in the distribution of TH^+^ cells in the midbrain, which suggested that Dkk1 might also play a role in morphogenetic movements in the midbrain. This possibility was confirmed by the severe alteration in the position of the few remaining midbrain DA neurons in the *Dkk1^−/−^* embryos and the severe morphogenetic defect. However, as these embryos lacked structures anterior to the midbrain, the observed phenotype can be indirectly contributed by additional factors. Previous reports have also shown that Dkk1 can modulate gastrulation movements independently of β-catenin and through activation of the Wnt/PCP pathway [22], a pathway that is required for proper neural tube closure [46]. Interestingly, 20% of the analyzed E13.5 *Dkk1^+/−^* embryos displayed exencephaly with a collapsed ventricle due a neural tube closure failure (data not shown), suggesting a possible modulation of the Wnt/PCP pathway by Dkk1 in the midbrain. In agreement with this possibility, exencephaly has also been described in *Lrp6* null embryos [31], [47]. In these animals, exencephaly was found to result from a disinhibition of Wnt/PCP signaling, which could be rescued by the loss of one or both *Wnt5a* alleles [47]. These results thus suggest that in addition to a balance within the Wnt/β-catenin signaling pathway, an intricate and complex cross-talk between the different branches of Wnt signaling, including the Wnt/PCP pathway, controls several aspects of neural development including morphogenesis.

In conclusion, our data show a novel role of Dkk1 in the differentiation of intermediate DA precursors into midbrain DA neurons and in midbrain morphogenesis.

## Supporting Information

Figure S1 (A) The expression of Tuj1 in *Dkk1^+/−^* embryos is not affected at any rostro-caudal level of the VM, at E13.5. Scale bar = 100 µm (B) No differences in the crown-rump length were observed in the analyzed embryos (mean ± s.e.m- ***Wt*** **:** 0. 97±0.03, N = 3; ***Dkk1^**+/−**^*** **:** 0.95±0.02, N = 12).
(TIF)

Figure S2 No alteration was detected in the number of Islet1^+^ cells in *Dkk1^+/−^* embryos (A,B) (mean ± s.e.m- ***Wt*** **:** 986.3±241.9, N = 3; ***Dkk1^**+/−**^*** **:** 1046±113.2 N = 5), or in the number of Brn3a^+^ cells (C,D) (mean ± s.e.m- ***Wt*** **:** 4973±121.0, N = 2; ***Dkk1*** **^**+/−**^:** 4783±269.5, N = 3). Scale bars = 100 µm.
(TIF)

Figure S3 (A) Very few active Caspase 3^+^ cells were detected in both *Wt* and *Dkk1^+/−^* embryos, and there were no differences in the number of positive cells (mean ± s.e.m- ***Wt*** **:** 1±1, N = 2; ***Dkk1*** **^**+/−**^:** 1.6±0.6, N = 3). (B) No changes in the numbers of Ki67+ cells were detected. Scale bar = 100 µm.
(TIF)

Figure S4 *Dkk1* expression was upregulated in mouse E10.5 VM primary cultures treated with Wnt3a for 6 hours (mean ± s.e.m- **BSA** **:** 0.322±0.008; **Wnt3a:** 0.667±0.008 N = 3, p = 0.002 ** paired t-test).
(TIF)
