# Supplementary figures and images for: Dkk1 Regulates Ventral Midbrain Dopaminergic Differentiation and Morphogenesis
Source: PLoS One. 2011 Feb 11;6(2):e15786. doi: 10.1371/journal.pone.0015786 (PMC3037958; doi:10.1371/journal.pone.0015786)

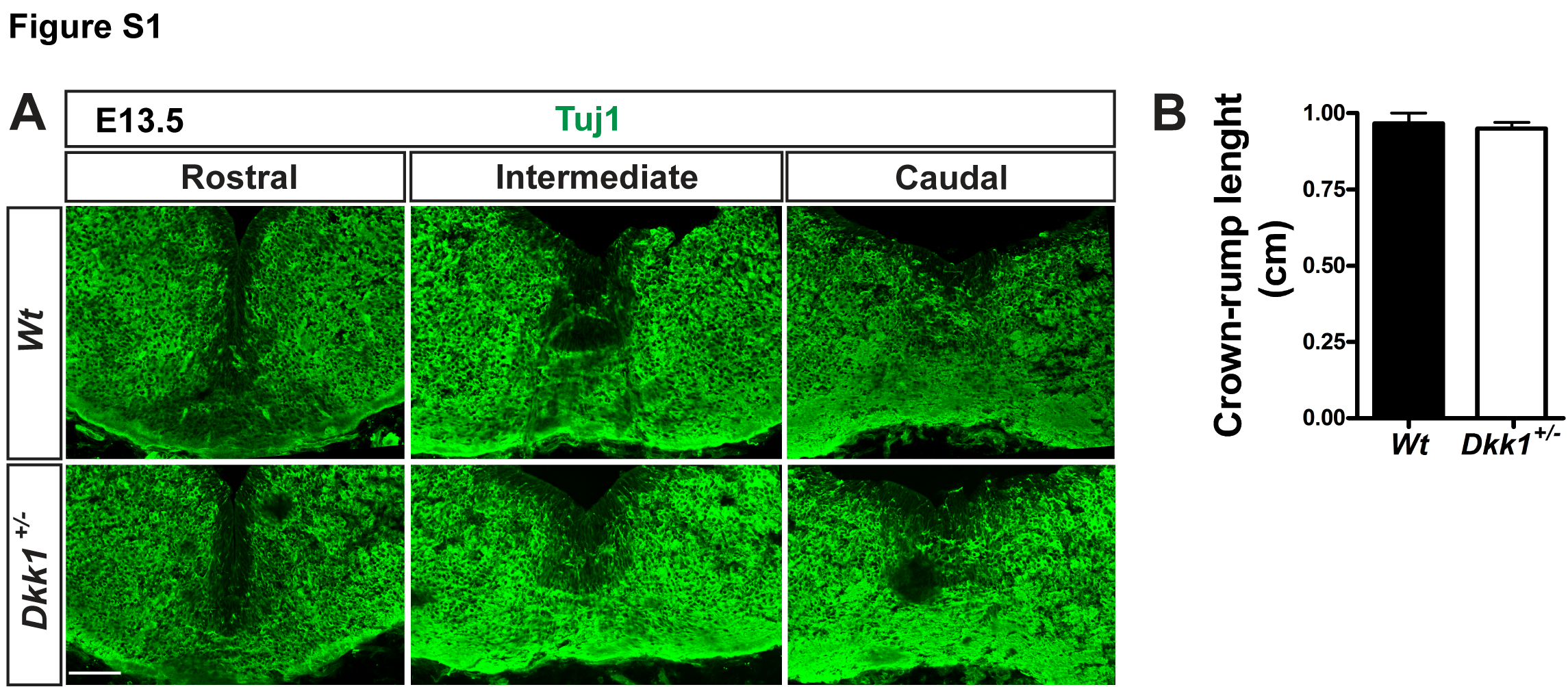

Supplement: Figure S1 — (A) The expression of Tuj1 in Dkk1+/− embryos is not affected at any rostro-caudal level of the VM, at E13.5. Scale bar = 100 µm (B) No differences in the crown-rump length were observed in the analyzed embryos (mean ± s.e.m- Wt : 0. 97±0.03, N = 3; Dkk1+/− : 0.95±0.02, N = 12). (TIF) [file pone.0015786.s001.tif]

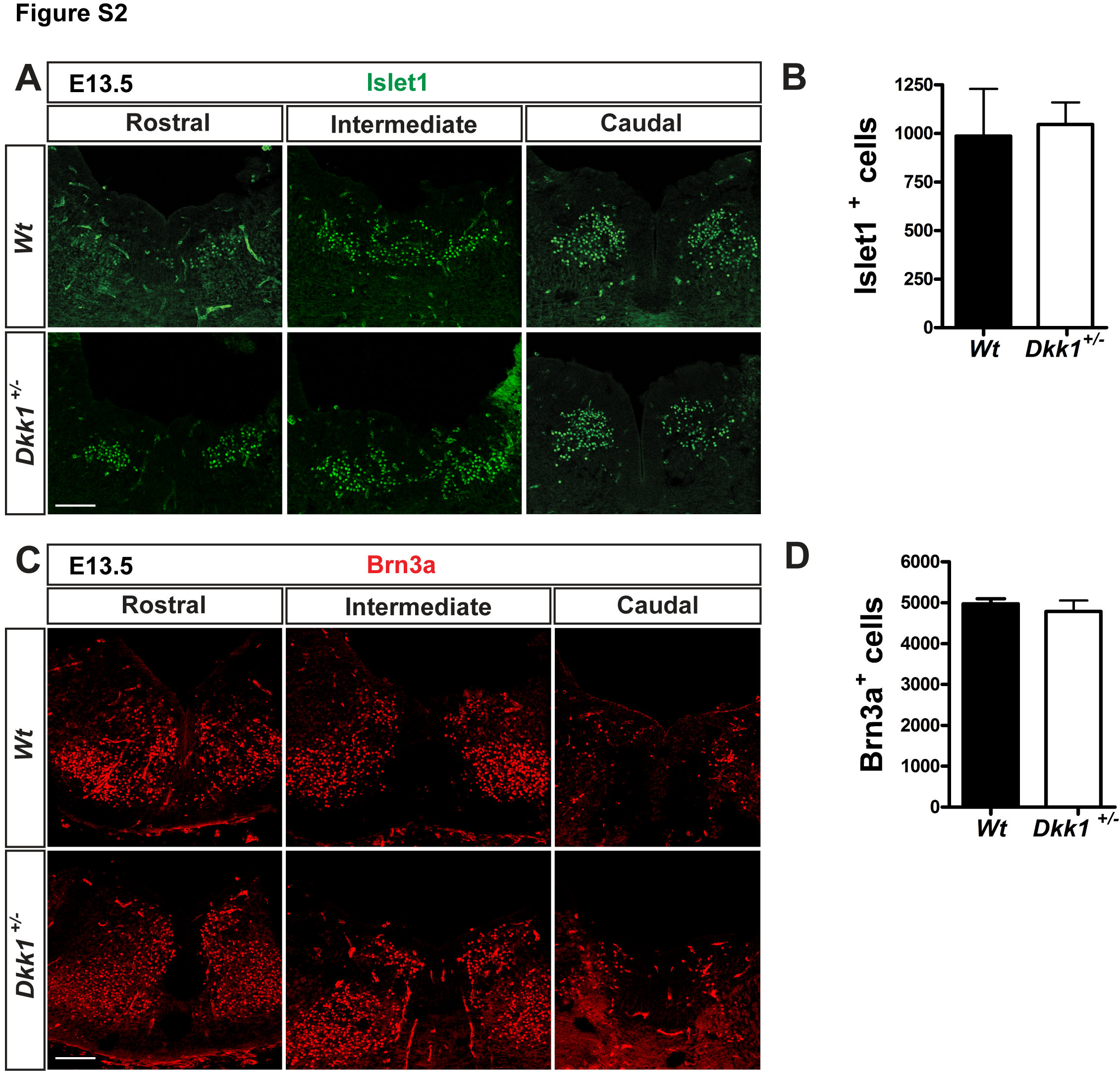

Supplement: Figure S2 — No alteration was detected in the number of Islet1+ cells in Dkk1+/− embryos (A,B) (mean ± s.e.m- Wt : 986.3±241.9, N = 3; Dkk1+/− : 1046±113.2 N = 5), or in the number of Brn3a+ cells (C,D) (mean ± s.e.m- Wt : 4973±121.0, N = 2; Dkk1 +/−: 4783±269.5, N = 3). Scale bars = 100 µm. (TIF) [file pone.0015786.s002.tif]

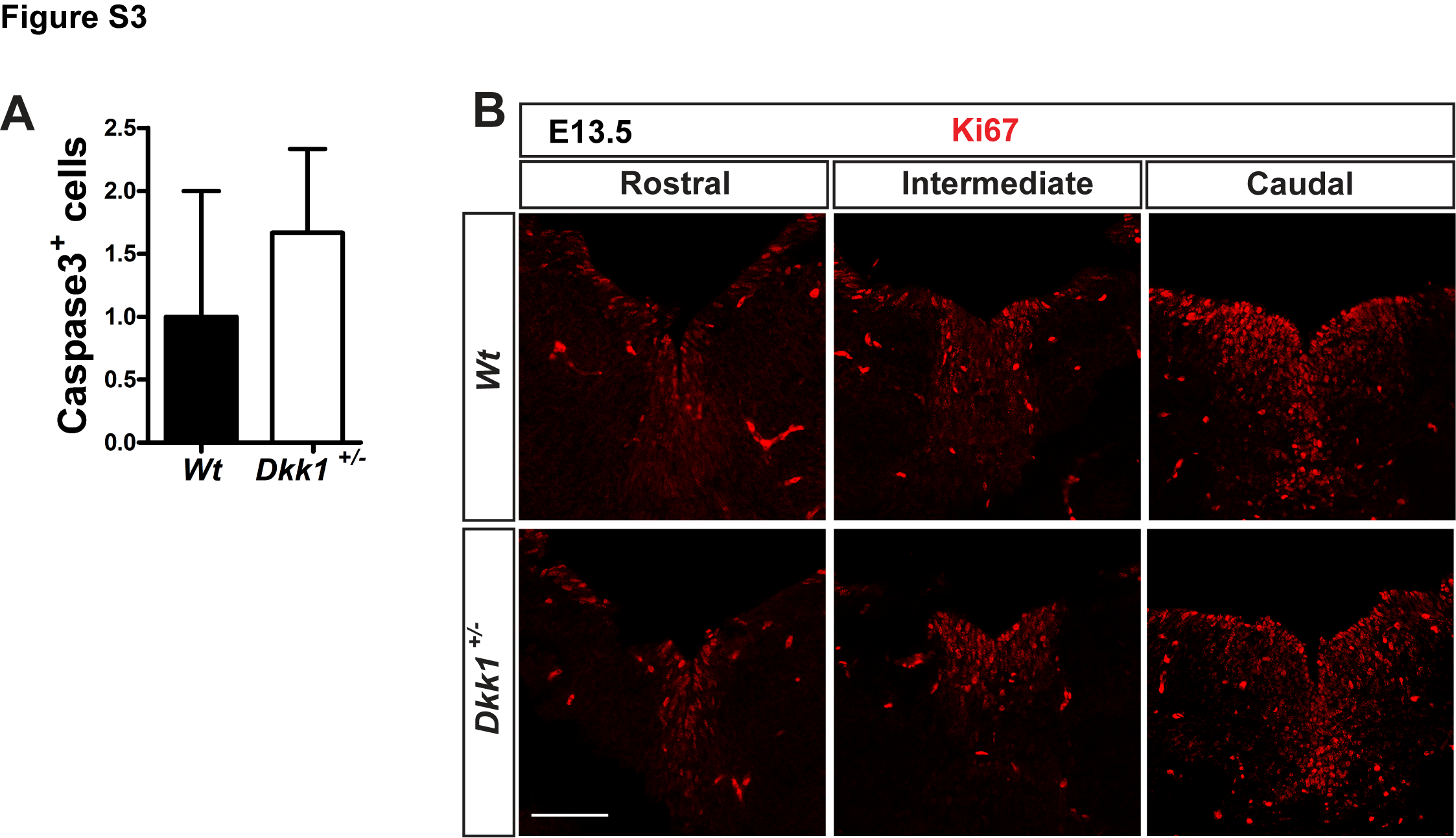

Supplement: Figure S3 — (A) Very few active Caspase 3+ cells were detected in both Wt and Dkk1+/− embryos, and there were no differences in the number of positive cells (mean ± s.e.m- Wt : 1±1, N = 2; Dkk1 +/−: 1.6±0.6, N = 3). (B) No changes in the numbers of Ki67+ cells were detected. Scale bar = 100 µm. (TIF) [file pone.0015786.s003.tif]

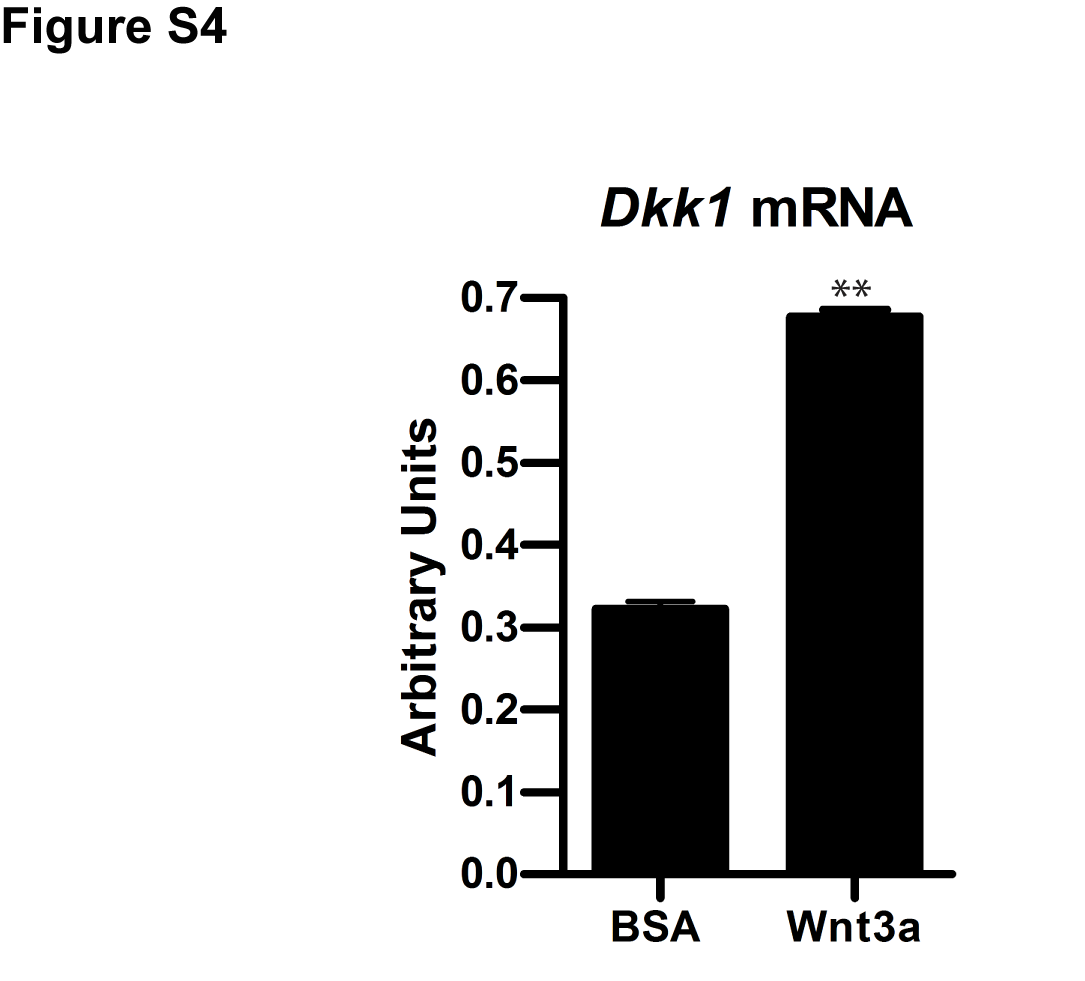

Supplement: Figure S4 — Dkk1 expression was upregulated in mouse E10.5 VM primary cultures treated with Wnt3a for 6 hours (mean ± s.e.m- BSA : 0.322±0.008; Wnt3a: 0.667±0.008 N = 3, p = 0.002 ** paired t-test). (TIF) [file pone.0015786.s004.tif]
